# Detection of Biological Bricks in Space. The Case of Adenine in Silica Aerogel

**DOI:** 10.3390/life9040082

**Published:** 2019-10-26

**Authors:** Aline Percot, Emilie-Laure Zins, Amélie Al Araji, Anh-Tu Ngo, Jacques Vergne, Makoto Tabata, Akihiko Yamagishi, Marie-Christine Maurel

**Affiliations:** 1 Département de Chimie Sorbonne Université, CNRS MONARIS UMR8233, 4 Place Jussieu, F-75005 Paris, France; emilie-laure.zins@sorbonne-universite.fr (E.-L.Z.); ameliearaji@msn.com (A.A.A.); anh-tu.ngo@sorbonne-universite.fr (A.-T.N.); 2 Institut de Systématique, CNRS Sorbonne Université, MNHN, UMR 7205, Evolution, Biodiversité, ISYEB, F-75005 Paris, France; jacques.vergne@mnhn.fr; 3 Department of Physics, Chiba University, Chiba 263-8522, Japan; makoto@hepburn.s.chiba-u.ac.jp; 4 Department of Applied Life Sciences, Tokyo University of Pharmacy and Life Sciences, Hachiojishi, Tokyo 192-0392, Japan; yamagish@toyaku.ac.jp

**Keywords:** adenine, Tanpopo mission, aerogel, SERS, origins of life

## Abstract

Space missions using probes to return dust samples are becoming more frequent. Dust collectors made of silica aerogel blocks are used to trap and bring back extraterrestrial particles for analysis. In this work, we show that it is possible to detect traces of adenine using surface-enhanced Raman spectroscopy (SERS). The method was first optimized using adenine deposition on glass slides and in glass wells. After this preliminary step, adenine solution was injected into the silica aerogel. Finally, gaseous adenine was successfully trapped in the aerogel. The presence of traces of adenine was monitored by SERS through its characteristic bands at 732, 1323, and 1458 cm^−1^ after the addition of the silver Creighton colloid. Such a method can be extended in the frame of Tanpopo missions for studying the interplanetary transfer of prebiotic organic compounds of biological interest.

## 1. Introduction

How and where did life originate? Water, carbon monoxide, sulfur dioxide, methane, and dinitrogen molecules present in the atmosphere of the primitive Earth might have reacted to form organogenic molecules, such as ammonia, hydrogen cyanide, formaldehyde, acetonitrile, cyanogen, and cyanoacetylene. Cosmic rays, ionizing reactions, electric discharges, mechanical and radioactive processes could have induced ion-molecule and radical reactions. From these molecules, the formation of the elementary building blocks of life, which are amino acids, nucleic acid bases, as well as polycyclic aromatic hydrocarbons and derivatives, could be explained [1,2,3,4]. These compounds may have been delivered on the primitive Earth by extraterrestrial dusts, comets, meteorites, etc. Delsemme emphasized that comet bombardments have brought to Earth all the carbon compounds present in organic molecules used by life [5].

On the other hand, Deamer [6,7], Deamer and Pashley [8], Oro et al. [4], and Vergne et al. [9], showed that carbonaceous chondrites contain a wide variety of molecules of biological interest. Following these pioneering discoveries, huge amounts of amino acids and of nitrogenous bases have been found and analyzed in meteorites [10]. 

Furthermore, nucleobases, in particular, the adenine ring, have a remarkable photo-stability in spite of their high absorption of ultraviolet (UV) radiation [11]. According to the importance of adenine in current biological processes and as a metabolic cofactor in ATP, ADP, AMP, NAD, NADP, FAD, CoA, cAMP, as well as in DNA and RNA, we were interested in adenine especially since we have a specific method for its detection.

Following our first work on the detection of adenine in meteorites by Raman spectrometry [12,13,14], we decided to develop a powerful analytical process for the identification of this elemental brick of nucleic acids within a matrix such as aerogel, a very low-density silica gel, embedded in the Tanpopo missions. Tanpopo missions started in 2015 on International Space Station (ISS) orbiting around the Earth at about 400 km above surface [15]. One of the scientific targets of Tanpopo missions is to test the space origin of organic compounds that contributed to the origin of life on Earth. Before the origin of life, organic compounds synthesized in space may have been transferred in micrometeorite. Silica aerogel block was used to capture micrometeorite in Tanpopo missions. By using super-low density, 0.01 g cm^−3^ silica aerogel, hypervelocity particles are expected to be captured with minimal denaturation. Silica aerogel blocks were contained in aluminum cases, capture panels, exposed to outer space on the ISS for capturing hyper-velocity particles including micrometeorites, and returned to the ground for analyses [15].

This work aims at setting up a procedure for the detection of potential traces of adenine in samples returning from Tanpopo missions. To this end, available blank aerogel samples were doped with adenine. Two complementary and distinct approaches were followed:

- Injections of solutions containing adenine;
- Exposure to adenine in the gas phase.

Adenine was then detected by surface-enhanced Raman spectroscopy (SERS). This method allows the main limitation of “classical” Raman spectroscopy, its low sensitivity, to be by-passed. In the SERS approach, the signal is enhanced by a nanostructured surface, often gold, copper, or silver. It has been demonstrated that this technique is well suited for adenine analysis in solution [16,17]. In our case, however, adenine is to be detected inside an inert matrix (aerogel), which represents an additional challenge for the analysis. 

First, we optimized the SERS detection of adenine with the silver Creighton colloid (incubation time, salt, and adenine concentration) using the deposit of traces on glass supports to mimic the effect of the silica aerogel [18]. Then this methodology was applied to the detection of adenine in aerogel samples after injection and exposure to adenine in the gas phase.

## 2. Materials and Methods

### 2.1. Raman Instrumentation

Raman spectroscopy measurements were performed using a LabRam HR800 (Horiba-Jobin Yvon, Ltd., Kyoto, Japan) instrument characterized by a focal length of 800 mm and edge filters. The analyses were done using the 458 nm excitation wavelength of a water-cooled Ar^+^ laser and a 600 lines/mm grating. The detector is a CCD camera with a Peltier effect cooling system. The spectral resolution was around 2 cm^−1^, and calibration was checked with respect to the 520 cm^−1^ silicon band. The spectrometer is coupled to an Olympus microscope equipped with a 50× Olympus objective, which allows a ~3 µm diameter spot size. The laser power was adjusted depending on the analyzed sample (nearly 12 mW at the sample for most of them), and the counting time was between 30 s to 1 min.

### 2.2. Chemicals

Adenine was purchased from Sigma. Adenine solutions were prepared in ethanol (95%); a stock solution at 10^−3^ M was prepared and diluted as necessary. The solutions have to be carefully checked before use, especially for concentrated solutions, because precipitation may occur. 

The silica aerogel is basically inorganic, resistant against the space environment, and makes it easy to analyze the hyper-velocity tracks because of its transparency. The aerogel block with the flight model quality was manufactured as described by Tabata et al. [19], accomplishing the minimal level of organics contamination. All the chemicals and solvents used were of the highest quality available. Aerogel blocks were manufactured under a contamination-controlled environment in the facility at Chiba University [19]. Wet silica gels were synthesized by the sol–gel method, aged, and rendered hydrophobic. Polymethoxy siloxane (silica precursor), aqueous ammonia (water source and catalyst), and solvent (ethanol) were mixed in the previously determined ratio [19]. Hexamethyldisilazane was used as the hydrophobic reagent. The wet gels, washed by ethanol, were dried by the supercritical carbon dioxide extraction method, obtaining silica aerogel blocks with a density of approximately 0.01 g cm^−3^.

Silver colloids were prepared by the slow addition of 100 mL silver nitrate solution (10^−3^ M) to 300 mL sodium borohydride solution (2 × 10^−3^ M) under vigorous stirring at low temperature (in an ice bath) in the dark [18]. The yellow solution obtained displayed an absorption band centered at 398 nm (measured on a Cary 3 UV-visible spectrometer).

### 2.3. Preparation and Characterization of Model Samples

First, 10 µL of adenine in ethanol (10^−3^ M) was totally evaporated on glass slides. Then, 90 µL of colloid and 10 µL of 500 mM MgCl_2_ were added into the same spot. Raman spectra were then registered as a function of time to monitor the SERS signal with time and water evaporation.

Second, to limit the heterogeneity under evaporation and to approach quantitative conditions, experiments were performed in glass wells of 2 mm diameter and 3 mm depth. The samples were prepared in the well using the following steps: addition of adenine/ethanol solution, evaporation of ethanol, addition of 3 μL of silver colloid (premix: 90 μL silver colloid, with 10 μL MgCl_2_, 500 mM). SERS spectra were recorded after 15 min incubation. The strong characteristic water band at 3425 cm^−1^ was used for intensity normalization. For the control experiment, 10 μL of pure ethanol was evaporated before the colloid mixture addition.

### 2.4. Preparation of Adenine-Containing Aerogel Samples

Adenine injection: controlled volume of adenine–ethanol solutions were injected in the aerogel via a Hamilton syringe. A small volume of 1 µL was injected to avoid diffusion of adenine inside the aerogel. 

Gaseous adenine: adenine powder (5 g) was heated in a glass tube (sand bath) at 120 °C under a stream of nitrogen, with bubbling in water. After 4 days, UV absorbance of water was measured to control adenine concentration. Next, aerogel was inserted in the tubing to force this adenine/nitrogen stream to pass through it.

### 2.5. SERS of Adenine in Aerogel

In the case of injection, colloid deposit and analysis were performed on the injection spot. For the aerogel submitted to gaseous adsorption, the aqueous colloid was deposited on a surface defect exposed to the gaseous adenine/nitrogen stream. Three to 5 μL of silver colloid (premix: 90 μL silver colloid, with 10 μL MgCl_2_, 500 mM) were added. The control was made in the same conditions on pristine aerogel. Focus was made on the aerogel/silver colloid interface at key points (using the C-H stretching mode of the aerogel around 2900 cm^−1^, and the O-H stretching mode of water molecules around 3425 cm^−1^). Spectra were registered on this focus as a function of time. The most significative spectra are presented at the end of the paper. These experiments were performed at least 3 times with high reproducibility.

### 2.6. Scanning Electron Microscopy (SEM) of Colloid and Salt Deposition on Aerogel

The colloid deposits were imaged with a JEOL model JSM-5510LV scanning electron microscope. The chemical compositions were determined by energy dispersive X-Ray spectrometry (EDS) analysis using a scanning electron microscope (SEM, JEOL 5510 LV, Ltd, Tokyo, Japan) with IXRF Systems 500 digital processing. Analyses were conducted on adenine-containing aerogel samples (prepared as previously described by ethanol-adenine injection) after the addition of silver colloid MgCl_2_ solutions and complete drying. 

## 3. Results and Discussion

### 3.1. A. Preliminary Remarks

It is worth underlining that the aerogel used in the Tanpopo missions has undergone a chemical treatment making it particularly hydrophobic [15,19,20]. This treatment prevents the deterioration of the structure of the aerogel during storage before and after the exposure experiment. The treatment also prevents the aerogel from overflowing with water, which would then prevent the trapping of further molecules of interest. 

This hydrophobicity constituted a challenge for the preparation of the specimen samples. Indeed, the injection of adenine solution was initially considered as a relevant approach for the introduction of a controlled amount of adenine into the aerogel. This approach proved impossible to implement with aqueous solutions, due to very little wetting of the aerogel by water: the drop introduced with a syringe into the aerogel was immediately rejected. Therefore, different organic solvents were tested for the introduction of adenine solutions into the aerogel using a syringe. This hydrophobicity problem also had to be overcome for SERS, since an aqueous solution must be used for the addition of the Creighton colloid and the salt solution. The use of a solvent that slightly alters the surface of the aerogel when injecting adenine proved to be the best alternative. Thus, the injection of ethanol adenine solution created a small well in the aerogel, in which it was further possible to introduce the Creighton colloid and the salt solution.

Due to the hydrophobicity of the aerogel, the adsorption of volatiles was not obvious. Nevertheless, it is interesting to evaluate the trapping of molecules in this 3D matrix for the study of certain environments, for example, in the case of degassing comets. This also concerns interplanetary dusts (IDPs), a source of organic volatiles. To this end, we forced a gaseous adenine streaming through the aerogel, simply by using its saturated vapor pressure, under slight heating and nitrogen flow.

### 3.2. B. Adenine Detection

**Figure 1:**
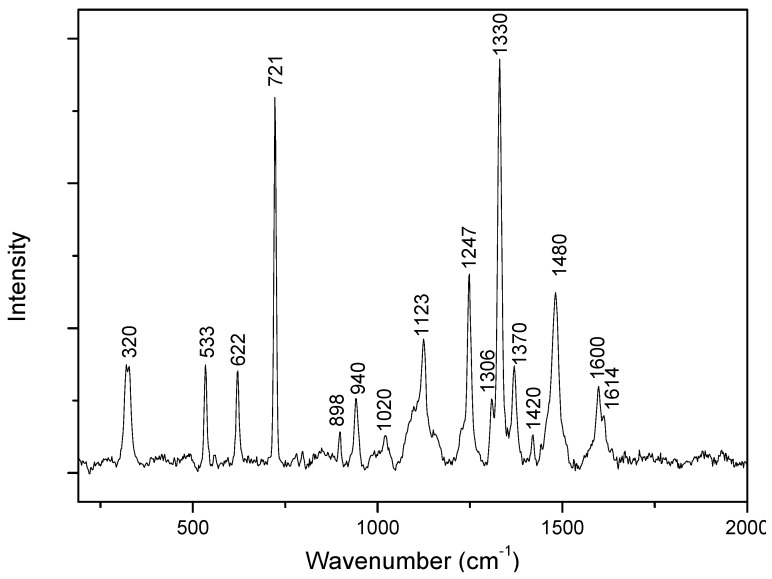
Raman spectrum of adenine powder.

Adenine has been the subject of numerous SERS studies since the early 2000s. However, there are some debates about how adenine adsorbs on a silver or gold surface, as well as its orientation, which are crucial parameters for the exaltation of the SERS signal [21,22,23,24,25]. There is also uncertainty about the exact assignments of some bands [16,17,26,27,28]. However, its SERS spectroscopic signature is well known. Figure 1 presents the Raman spectrum of evaporated adenine-ethanol powder. The main attributions based on data available in the literature are summarized in Table 1.

**Figure 2:**
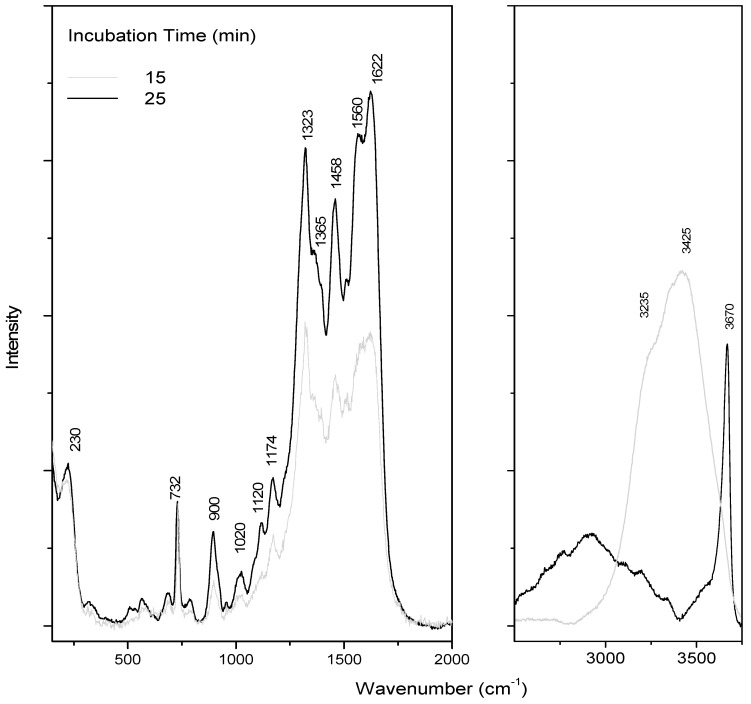
SERS adenine spectra as a function of incubation time. A colloid drop was added on dried adenine (10 µL of adenine–ethanol solution at 10^−3^ M) on a glass slide.

SERS is a kinetic-sensitive process. The adsorption of adenine on the colloid takes some time, needs salt, and is sensitive to water evaporation (and concentration changes) [21,30]. All these processes affect the orientation of adenine, which will directly influence its SERS response, inducing drastic changes in the spectra as a function of time and drying, as already pointed out in the literature [16]. Moreover, in our case, adenine is trapped in the aerogel lattice, which also perturbs the adenine adsorption. The concentration effect is illustrated in Figure 2, where spectra were registered as a function of incubation time. For these experiments, a colloid drop was added on dried adenine on a glass slide.

A first SERS pattern was observed after 15 min under laser, showing intense bands at 732, 1323, and 1458 cm^−1^, attributed to adenine (Table 1). As previously mentioned, exaltation is a complex phenomenon, sensitive to numerous parameters. In this study, we chose to register spectra after 15 min incubation (when water was still present, as shown by the band at 3425 cm^−1^). The band at 732 cm^−1^ was assigned to a breathing mode, and the multi-component band at 1323 cm^−1^ was assigned to mixed in-plane stretching motions of the six-membered ring. 

On this spectrum, a large band due to water was still observed around 3425 cm^−1^ (Figure 2). A decrease of the water band intensity was observed within the time and corresponds to the drying process. After drying, the same characteristic SERS pattern was observed. The band at 230 cm^−1^ is associated with chloride adsorption. Indeed, MgCl_2_ was added to optimize adenine detection, by recording SERS spectra in solution as a function of salt concentration (data not shown). The final chosen MgCl_2_ concentration was 50 mM. 

Despite these discrepancies in the SERS signatures of adenine (Figure 2), the same features were reproduced during the drying of the mixture.

**Figure 3:**
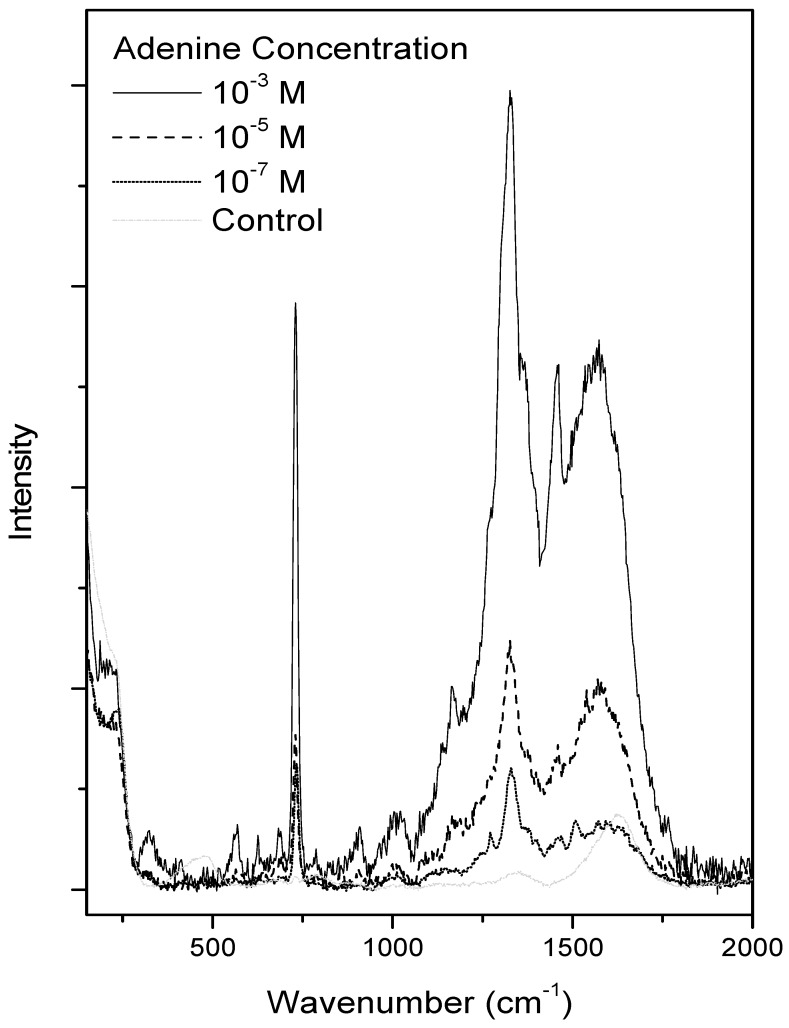
SERS adenine spectra as a function of adenine concentration in glass wells after 15 min incubation, with control spectrum (colloid mixture).

During drying, a gravitational force induced a non-homogeneous deposition of adenine and silver colloid on the glass slide. Glass wells were further used to limit the so-called “coffee ring” phenomenon that caused 2D inhomogeneity of analytes, nanoparticles, and salt [30]. Furthermore, this series of experiments is a first step towards the understanding of the analysis of the SERS adenine in the aerogel. The spectra thus obtained are presented in Figure 3. For the sake of comparison, all spectra were registered after 15 min incubation. One microliter of adenine/ethanol solution at 3 different concentrations was used, namely 10^−3^, 10^−5^, and 10^−7^ M. In all spectra, the characteristic spectral pattern with intense bands at 732, 1323, and 1458 cm^−1^ was observed. As expected, the more concentrated sample led to the more intense spectrum, despite the non-quantitative aspect of the SERS signal. In this region, the control spectrum presented only a broad band at 1600 cm^−1^ attributed to OH bending.

After this validation phase of the detection of adenine deposited in alcoholic solution, we sought to detect adenine in aerogel. One microliter of adenine solution was locally injected into the aerogel. A morphologic change was observed, with a local whitening of the aerogel. As already underlined, this morphologic defect (due to chemico-physical changes in aerogel with ethanol) made it easier to add aqueous colloids and salt on the adenine deposition.

**Figure 4:**
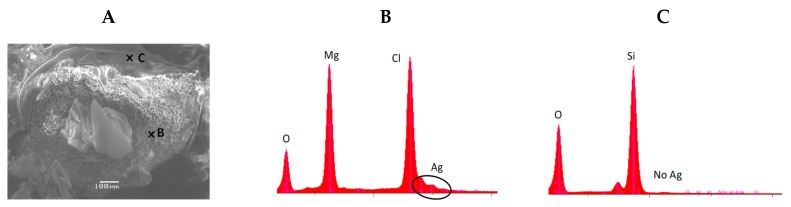
SEM image of the aerogel after adenine and colloid and salt deposit and drying (**A**). (**B**) and (**C**) energy dispersive X-Ray spectrometry (EDS) analysis on the focus B and C, respectively.

**Figure 5:**
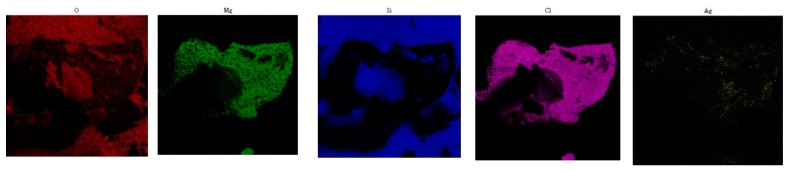
Cartographies showing the 2D distribution of some elements characteristic of the colloid and salt (Mg, Cl, Ag) and aerogel (O, Si) (Figure 4A).

As in the case of the SERS analyses, alcoholic solutions of adenine were first injected into the aerogel before colloid and salt mixture deposit. To ascertain the co-deposition of the colloid and salt solutions on this hydrophobic surface, SEM and EDS analyses were carried out on the aerogel (Figure 4 and Figure 5). 

Figure 4A shows a SEM image of the aerogel after drying. Figure 4B,C show the EDS spectra taken on the focus B and C, respectively. On the spectrum 4B, the magnesium, chloride, and silver signatures were observed. This proves the presence of the silver colloid and salt on the aerogel (despite hydrophobicity). On focus C, only the signature of the aerogel was observed. Figure 5 demonstrates the co-localization of magnesium, chloride, and silver on the aerogel. 

It, therefore, seems entirely appropriate to use this aqueous silver colloid for the SERS adenine detection on the aerogel.

**Figure 6:**
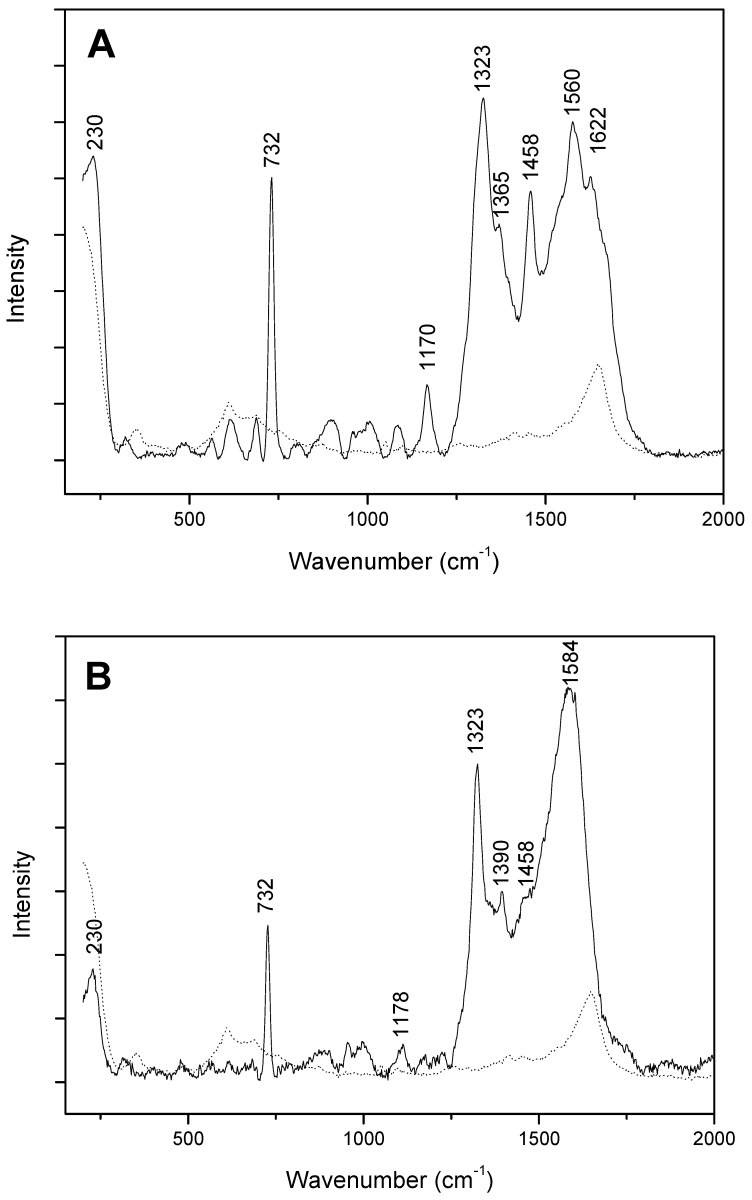
SERS adenine spectra after injection in aerogel (**A**) and adsorption from the gas phase (**B**) with control spectrum (aerogel and colloid mixture) (dots).

Figure 6A presents the SERS spectrum obtained after adenine injection of 10^−9^ mol in the aerogel. The spectrum was registered at the colloid interface before complete water drying. As expected under such conditions, spectrum with the spectral pattern presented in Figure 2 was observed. The control spectrum shows 2 broad bands at 610 and 1600 cm^−1^, which can be respectively attributed to silica aerogel and OH bending.

Finally, we investigated whether aerogel could also trap adenine in the gas phase. A nitrogen flow was introduced into a vessel containing adenine and placed on a sand bath maintained at 120 °C. A part of the adenine vaporized and was dragged by the nitrogen flow. Without the aerogel, the gaseous flow bubbled in water. After four days, the UV spectrum of the solution at 260 nm (absorbance above 1) and the SERS spectrum confirmed the presence of adenine (spectra not shown). After this validation of the process, the gas phase was forced to pass through a cotton filter before passing through the aerogel. After four days, the aerogel was recovered and analyzed by SERS. The colloid drop was deposited in a native aerogel asperity. The characteristic SERS feature of the adenine thus obtained (Figure 6B) demonstrates that the aerogel can adsorb volatiles such as adenine. The analyses were only carried out at the surface.

## 4. Conclusions

The identification of prebiotic molecules from extraterrestrial origin remains a Holy Grail for astrochemists and astrophysicists. Our results show that the aerogel developed for the Tanpopo missions efficiently traps adenine. The use of the SERS technique with this hydrophobic matrix is delicate since the silver colloid interaction is dramatically affected by the drying. Despite this difficulty, the SERS analysis of the adenine on the aerogel remains possible, since the colloid and the salt can be deposited on the aerogel, as shown by the SEM and EDS analyses. 

The exalted vibration modes of adenine were disturbed by drying, even in the absence of aerogel, as seen with glass wells, probably due to a fundamentally different orientation of this aromatic molecule on these siliceous surfaces. However, in the presence of water, the spectroscopic signature found by SERS of adenine in the aerogel was similar to the spectra obtained in wells. 

Thus, the Creighton colloid seems quite relevant for the search for adenine in return samples from the Tanpopo missions. These promising results will be complemented by a study using three distinct and complementary approaches:

(i) It will be interesting to insert adenine-doped interstellar dust analogs into the aerogel and to detect adenine in it.
(ii) Complementarily, it would be interesting to develop other analytical techniques for the detection of adenine in this 3D matrix and its quantification.
(iii) From a more fundamental point of view, a comprehensive study on the parameters governing the orientation of adenine in relation to the colloid, and thus the excited vibration modes, would be a very beneficial point, which could allow the SERS technique to be optimized.

Over the longer-term, the miniaturization and automatization of Raman instruments and the control of SERS analysis could make it possible to consider taking onboard device during a space mission, and SERS analyses might be carried out in situ and in real-time [31]. 

## Figures and Tables

**Table 1:** Main bands observed in Raman and surface-enhanced Raman spectroscopy (SERS) spectra with their assignments.

Raman (cm^−1^)	SERS (cm^−1^)	Assignments	
		Ref. [16,26,28,29]	Ref. [17]
320		bend C6-NH_2_	
533		def R6, R5 wag N9-H	
622		def R6, R5	ring bend
721	732	ring breath	ring breath
898	900	def R6, R5	
940		def R5	
1020	1020	rock NH_2_	
1123	1120	str C8-N9, A bend N9-H, C8-H	
	1174	str C6-N10, N3-C4, C4-N9, bend C8-H, N10-H11	
1247		bend C8-H, N9-H, str N7-C8	H bend, ring str
1306		str C2-N3, N1-C2, C5-C6, C5-N7	
1330	1323	str C5-N7, N1-C2, bend C2-H, C8-H bend C2-H, C8-H, N9-H, str C6-N1, C8-N9, N3-C4	H bend, ring str
1370	1365	bend C2-H, N9-H, str C8-N9, C4-N9	H bend, ring str
1420		str C4-N9, C4-C5, C6-N10, N7-C8, bend C2-H	H bend, ring str
1482	1458	str C2-N3, N1-C6, bend C2-H, sciss NH_2_	H bend, ring str
1600		sciss NH_2_	
1614	1622	str N3-C4, N1-C6, C5-N7, N7-C8, bend N9-H	

The following abbreviations have been used: bend, bending; breath, breathing; def, deformation; rock, rocking; sciss, scissoring; str, stretching; wag, wagging; R5, five-membered ring; R6, six-membered ring.
